# Primary Leiomyosarcoma of the Male Breast

**DOI:** 10.4021/wjon238w

**Published:** 2010-11-02

**Authors:** Daniel Boehm, Ksenia Keller, Marcus Schmidt, Christina Cotarelo, Annett Kern, Antje Lebrecht, Heinz Koelbl

**Affiliations:** a Department of Obstetrics and Gynaecology, University Medical Center Mainz, Langenbeckstr. 1, 55131 Mainz, Germany; b Department of Pathology, University Medical Center Mainz, Langenbeckstr. 1, 55131 Mainz, Germany; c Department of Radiology, University Medical Center Mainz, Langenbeckstr. 1, 55131 Mainz, Germany

**Keywords:** Leiomyosarcoma, Male breast, Mastectomy

## Abstract

We describe herein the third case of primary leiomyosarcoma of the breast in a 62-year-old man. Preoperative clinical examination and cytology findings indicated a leiomyosarcoma of the breast. A modified radical mastectomy was performed. Immunohistochemical analysis subsequently confirmed a diagnosis of leiomyosarcoma. After a follow-up period of 24 months, the patient is still in good health with no evidence of locoregional recurrence or distant metastasis.

## Introduction

Leiomyosarcomas of the breast are a rare subgroup of primary breast sarcomas. Even rarer is a primary leiomyosarcoma of a male breast. To date, only 3 cases have been reported in the literature.

Leiomyosarcoma of the breast is difficult to diagnose preoperatively; however, a correct histopathologic diagnosis is important for determining the most appropriate surgical procedure. This report describes a case of primary leiomyosarcoma of the breast in a 62-year-old man. We describe the clinical features, diagnosis, and treatment of primary leiomyosarcoma of the male breast with a literature review. The purpose of this work is to increase physicians' awareness of such lesions.

## Case Report

A 62-year-old male patient was admitted to our hospital presenting a painless right breast mass that had been enlarging over the past three months. The patient's past medical history was without pathological findings. His family history was not significant for malignancies in any first-degree relatives.

**Figure 1:**
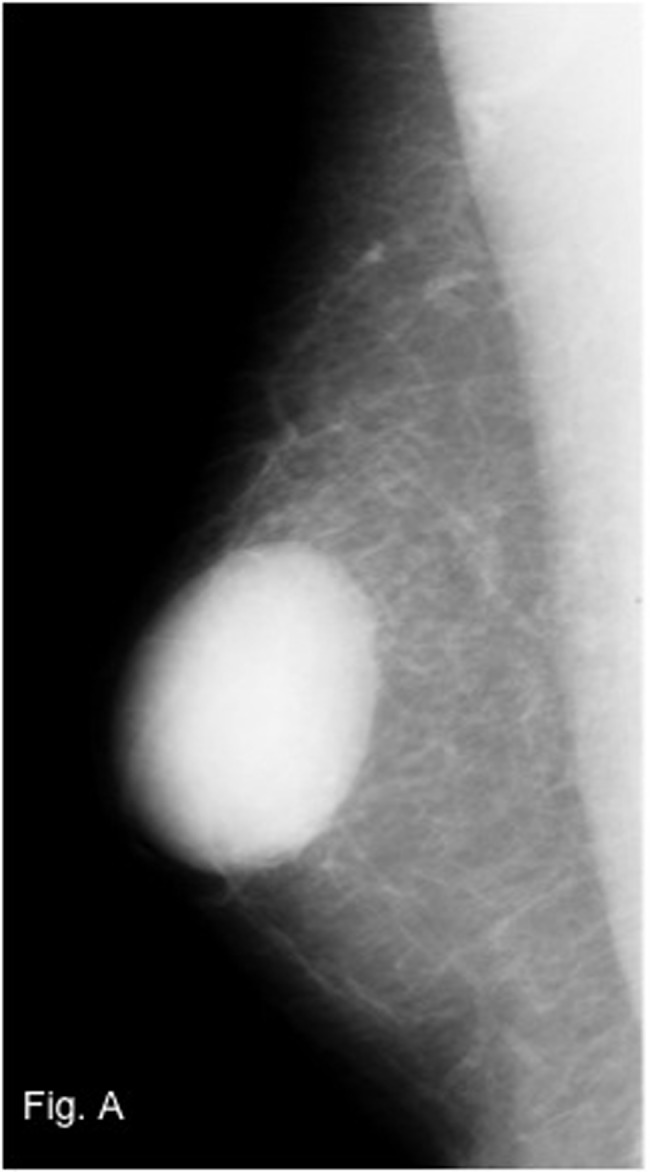
Mammography of the right breast with a dense mass in a central localization.

**Figure 2:**
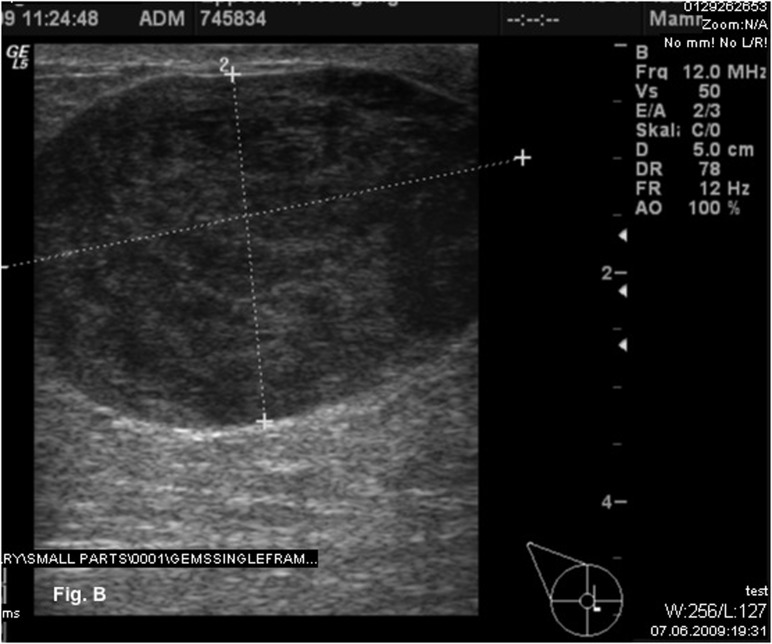
Ultrasonography showed a hypoechoic solid mass suggestive of a fibroadenoma.

Physical examination indicated a firm palpable mass in the area of the nipple-areola complex. No suspicious cervical, supraclavicular or left axillary lymph nodes were palpable. Mammography revealed a dense, well-circumscribed solid mass of 4.6 x 3.5 cm in a central localization of the right breast (Fig. 1). The tumor was assessed as BIRADS category 3. Subsequent ultrasonography showed a hypoechoic solid echotexture that was suggestive of a fibroadenoma (Fig. 2). Preoperative core biopsy of the tumor revealed a leiomyosarcoma. Bone scintigraphy and computertomography of the chest and abdomen detected no distant metastasis.

**Figure 3:**
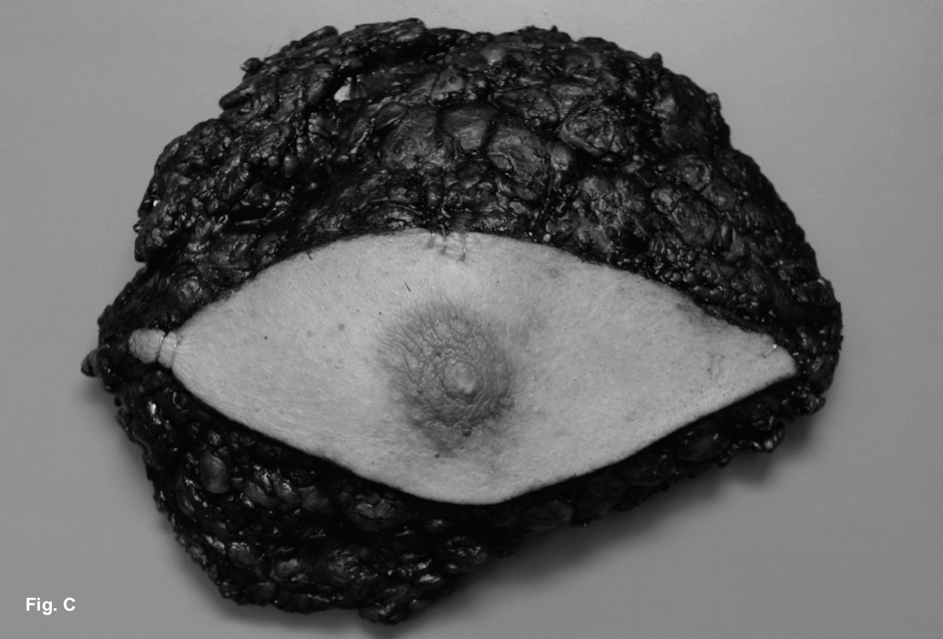
Modified radical mastectomy of the right breast was performed.

**Figure 4:**
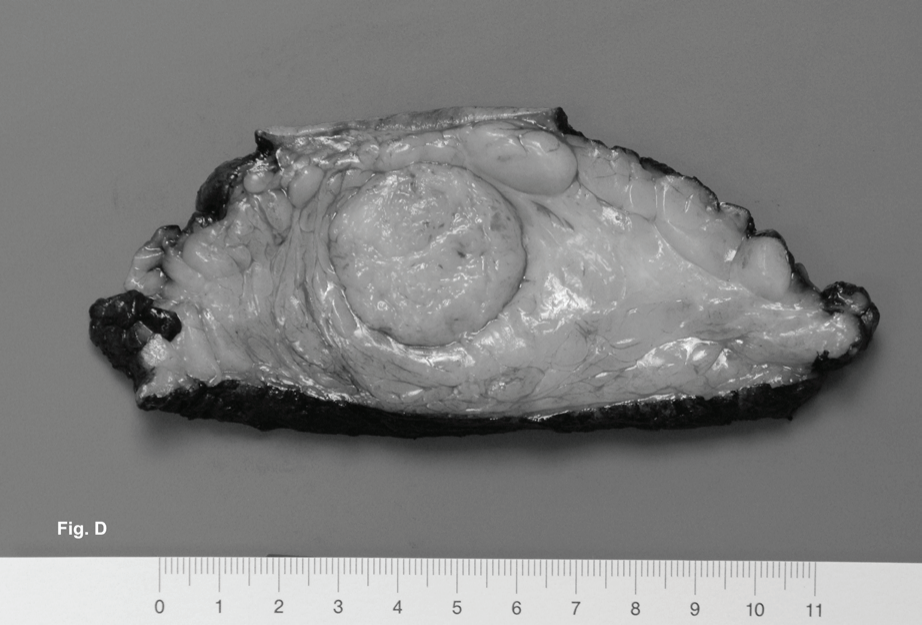
The tumor had a pale cut surface with sharply demarcated margins surrounded by fatty tissue.

**Figure 5:**
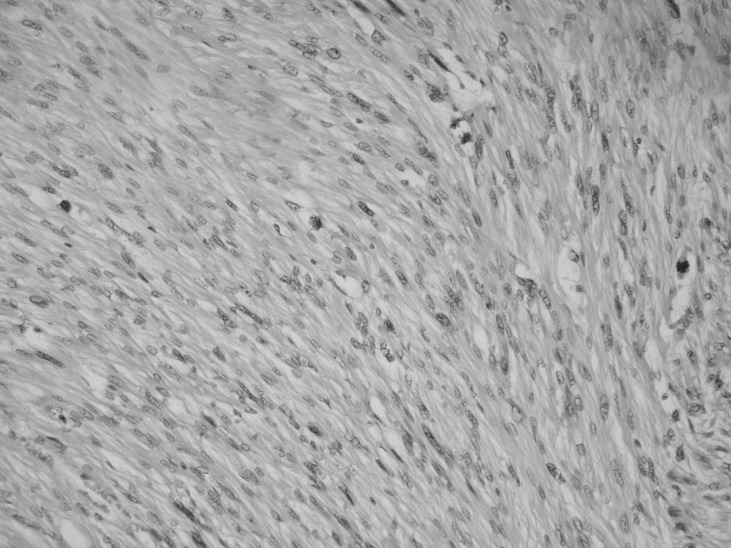
Microscopically, the leiomyosarcoma shows a spindle cell pattern.

A modified radical mastectomy of the right breast was performed (Fig. 3). Macroscopically, the tumor measured 4.4 x 3.4 x 3.4 cm and had a soft pale cut surface with sharply demarcated margins surrounded by fatty tissue (Fig. 4). Histological examination revealed a leiomyosarcoma of medium grade malignancy (4 mitoses per 1 high-power field). All surgical margins were negative for tumor. The malignant mesenchymal tumor showed predominant a spindle cell pattern (Fig. 5). Immunhistochemistry showed strong positivity for desmin and caldesmon. The cell proliferation marker Ki-67 showed a low expression of 5-10%.

After surgery the patient received chest-wall radiation therapy to a dose of 50 Gy during 6 weeks. After a follow-up period of 24 months the patient is still in good health with no evidence of locoregional recurrence or distant metastasis.

## Discussion

We reported an unusual case of leiomyosarcoma of the male breast. Sarcomas of the breast are rare, accounting for just 1% of all malignant breast tumors, among these primary leiomyosarcoma is an extremely rare type of breast sarcoma.

This is the fourth reported case of a primary leiomyosarcoma of a male breast. They were described before by Crocker et al 1969, Hernandez F. 1978 and Farkas et al 1991. There are 20 further cases of female patients. Due to the few cases published by now, there are only few clinical data available addressing optimal treatment modalities. The optimal surgical therapy recommendation is a complete excision with wide margins. Axillary dissection, chemotherapy and radiotherapy have not improved disease free or overall survival.

## References

[1] Crocker DJ, Murad TM (1969). Ultrastructure of fibrosarcoma in a male breast. Cancer.

[2] Hernandez FJ (1978). Leiomyosarcoma of male breast originating in the nipple. Am J Surg Pathol.

[3] Farkas E, Koves I, Besznyak I, Sapi Z, Sulyok Z (1991). [Leiomyosarcoma in the male breast]. Orv Hetil.

